# Apparent prevalence of transplacental transmission of hemotropic mycoplasmas in Holstein dairy calves

**DOI:** 10.3168/jdsc.2023-0518

**Published:** 2024-03-08

**Authors:** L. de Souza Ferreira, S. Bolin, A. Abuelo, B. Norby, P.L. Ruegg

**Affiliations:** 1 Department of Large Animal Clinical Sciences, College of Veterinary Medicine, Michigan State University, East Lansing, MI 48824; 2 Veterinary Diagnostic Laboratory, Michigan State University, Lansing, MI 48910

## Abstract

Hemotropic mycoplasmas are unculturable bacteria that infect the surface of red blood cells of several mammalian species including cattle. The importance of hemoplasmas in cattle remains unclear and limited information is available about biological routes of transmission. The objective of this cross-sectional study was to determine the prevalence of transplacental transmission of hemoplasmas and to determine if colostrum of infected cows contained DNA from hemoplasma organisms. In March 2023, researchers collected colostrum and peripheral blood samples from 39 dairy cows and their newborn calves (before ingestion of colostrum) at a single dairy farm in Michigan. Detection of *Mycoplasma wenyonii* and *Candidatus Mycoplasma haemobos* was performed using real-time PCR. The apparent prevalence of hemoplasma infection in dams was 100%, with 84.6% (33/39) co-infected with both *M. wenyonii* and *C. M. haemobos*, whereas the remaining 15.3% (6/39) were infected solely with *C. M. haemobos*. The prevalence of newborn calves infected with *C. M. haemobos* was 10.2% (4/39), and none were infected with *M. wenyonii*. No colostrum samples tested positive for either *M. wenyonii* or *C. M. haemobos*. This is the first report of vertical transmission of hemoplasmas in a dairy herd located in the United States. However, the relatively low prevalence of infected newborn calves suggests that transplacental transmission is not the predominant pathway of infection. The absence of positive tests in colostrum may indicate ingestion of colostrum contaminated with hemoplasmas is not common. The clinical importance of fetal infection remains known.

## Highlights

• Samples were collected from 39 dairy cows and their newborn calves from a dairy farm.
• All cows were positive for *C. M. haemobos* or were co-infected with *C. M. haemobos* and *M. wenyonii*.
• Of calves, 10.2% tested positive for *C. M. haemobos* in blood collected before ingestion of colostrum.
• DNA of *C. M. haemobos* or *M. wenyonii* was not detected in colostrum.
• Transplacental transmission may contribute to the spread of bovine hemoplasmas but is likely a minor pathway of infection.

## Graphical Abstract

**Figure:**
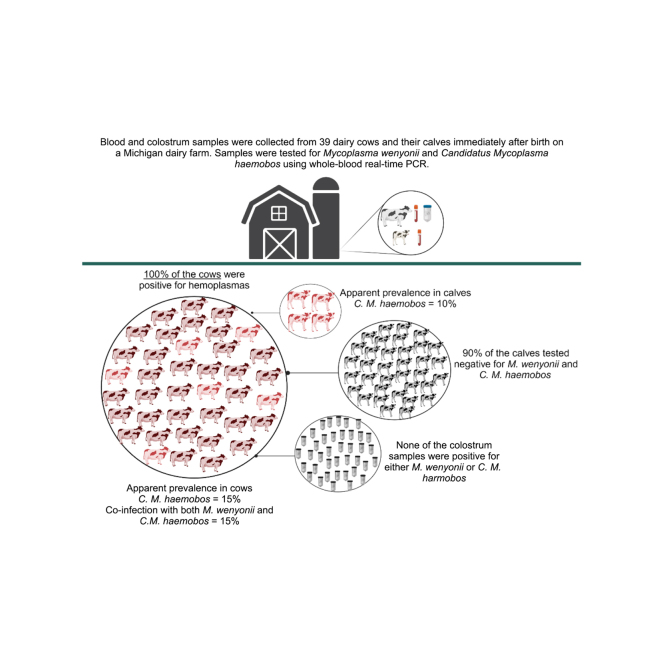
**Summary:** Colostrum and blood samples were collected from 39 dairy cows and their newborn calves from a single farm in Michigan. Samples were tested for *Candidatus Mycoplasma haemobos* and *Mycoplasma wenyonii* using whole-blood real-time PCR. This is the first report of vertical transmission of hemoplasmas in a Michigan dairy herd, suggesting that transplacental transmission could serve as an alternative route for the spread of bovine hemoplasma, but likely as a minor pathway of infection. There was no evidence of hemoplasma present in colostrum.

*Mycoplasma wenyonii* and *Candidatus Mycoplasma haemobos* are gram-negative, wall-less bacteria that have been described as major hemotropic mycoplasmas (hemoplasma) in cattle (Neimark et al., 2002; Tagawa et al., 2008). Hemoplasmas have been identified in blood from both symptomatic and apparently healthy cattle. Clinical signs of hemoplasma infection have included immune-mediated anemia (Gladden et al., 2016), anorexia (Genova et al., 2011), mammary gland edema (Smith et al., 1990), edema of rear legs (Genova et al., 2011), fever (Smith et al., 1990), prefemoral lymphadenopathy (Smith et al., 1990), reduced milk yield (Hoelzle et al., 2011; Strugnell and McAuliffe, 2012; Gladden et al., 2016), weight loss (Genova et al., 2011), and infertility (Montes et al., 1994). Although most infected cattle appear apparently healthy, the factors that trigger manifestation of clinical signs are not yet fully understood. When animals exhibit clinical signs, oxytetracycline has been reported to result in resolution of signs in some cattle (Montes et al., 1994; Genova et al., 2011). In the past, hemoplasma infections were diagnosed with observation of organisms on Giemsa-stained blood smears, but this method has low diagnostic accuracy and PCR testing is the preferred method of diagnosis (Tasker et al., 2018).

There is very little information about potential routes of transmission and risk factors for infection with hemoplasma in cattle. It is thought that transmission occurs through direct contact with infected blood or through blood-sucking insects (Smith et al., 1990; Fujihara et al., 2011). Biological modes of transmission, such as transplacental transmission, have been demonstrated in a few studies conducted in Hungary, Japan, Brazil, and Bavaria (Hornok et al., 2011; Girotto-Soares et al., 2016; Niethammer et al., 2018; Sasaoka et al., 2015). However, variations in the study population, such as differences in animal breeds, location, and the selective detection of organisms in certain studies (focusing on either *M. wenyonii* or *C. M. haemobos* instead of both organisms) make it challenging to fully characterize this transmission route. Ingestion of colostrum is another potential route of infection. To date, only a single study (Sasaoka et al., 2015) that focused on beef cattle explored colostrum as a potential source of infection. In that study, DNA of *M. wenyonii* was not detected in any of 17 colostrum samples assessed. To date, no studies have examined the occurrence of both *M. wenyonii* and *C. M. haemobos* in colostrum collected from dairy cows.

The objective of our study was to determine if infection with *M. wenyonii* or *C. M. haemobos* occurs before birth (vertical transmission) and to evaluate the presence of bovine hemoplasma DNA in the colostrum. We hypothesized that most infections with these organisms occur after birth and that transplacental transmission is uncommon.

The study was designed as a prospective cross-sectional study conducted in a single herd, with animals as the experimental unit. Prevalence of hemoplasma infection in youngstock of the herd used in this study had been previously determined to be >50% (unpublished data) and the herd was expected to have at least 35 calves born during the period selected for sampling. Researchers visited the farm during weekdays from 0800 to 1700 h during 2 wk in March 2023. All cows that calved during that period and their newborn calves were eligible for sampling, regardless of parity, breed, or sex. Study personnel observed parturition and collected whole blood samples from the coccygeal vessels of cows, and from jugular vein of their calves immediately after birth and before ingestion of colostrum using 10-mL serum-separator and EDTA tubes. Colostrum samples were collected from cows immediately after calving, following guidelines for aseptic sampling (NMC, 2017). After sampling, blood and colostrum samples were immediately cooled to 4°C for transport to the Michigan State University's Veterinary Diagnostic Laboratory (Lansing, MI) to undergo real-time PCR (**rt-PCR**) testing. The age and parity of each cow were obtained from herd records. Based on a standard formula to estimate sampling required for detection of disease (Dohoo et al., 2009), we estimated that sampling at least 15 calves was required to be reasonably confident that we would detect the disease if the prevalence was 1% or higher. This study was approved by the Institutional Animal Care and Use Committee at Michigan State University (Study ID 0008451).

Whole blood was collected into evacuated tubes containing EDTA and stored at 4°C for 24 to 48 h before DNA extraction. A magnetic bead assisted DNA extraction method was performed from 200 µL of whole blood using the KingFisher Flex Purification System (Thermo Fisher Scientific, Waltham, MA) with the MagMAX Core Nucleic Acid Purification Kit following the manufacturer's instructions. Real-time quantitative PCR assays were employed to detect and quantify DNA from *M. wenyonii* and *C. M. haemobos* using specific primers and probes targeting the 16S ribosomal RNA gene. The PCR reaction mixture for both organisms included 10 µL of 2× PerfeCTa qPCR ToughMix Low Rox (Quanta Bio, Beverly, MA), 400 nmol each of forward and reverse PCR primers, and 250 nmol of hydrolysis probe. For *M. wenyonii*, the forward primer was 5′-GAAAGYCTGATGGAGCAATA-3′, with a predicted melting temperature (**Tm**) range of 56°C to 59°C, and the reverse primer was 5′-SCTTTACGCCCAATAAATC-3′, with a predicted Tm range of 55°C to 56°C. The hydrolysis probe for *M. wenyonii* was JOEN-CGCGCCTTG/ZEN/ATGGTACTAATTGA-3IABkFq, with a Tm of 66°C. For *C. M. haemobos*, the forward primer was 5′-ACGAAAGTCTGATGGAGCAATAC-3′, and the reverse primer was 5′-ACGCCCAATAAATCCGRATAATG-3′. The hydrolysis probe for *C. M. haemobos* was FAM-TGAGGTACT/ZEN/ATCAGTTGTTATCCCTC-3IABkFq, with a predicted Tm of 65°C. Molecular grade water was added to the reaction mix to bring the reaction mix volume to 18 µL. Positive controls for PCR assays were synthetic DNA, and standard curves were generated from 10-fold dilutions of known DNA concentrations ranging from 10^2^ to 10^7^ copies/µL, tested in duplicate. The threshold for positive results was set at cycle threshold (**Ct**) <35.4. The negative control was reagent mix without any additions. Because the hemoplasmas cannot be cultured, the usual gold standard for sensitivity of colony-forming units is not available. Using synthetic DNA, the sensitivities of the PCR assays were less than 10 copies of DNA per reaction. The specificity for the PCR reactions was assessed by nucleic acid sequencing of the amplicons from the in-house gel-based confirmatory PCR assay. To confirm suspect results, an in-house diagnostic gel-based PCR assay was used. Precautions were taken to prevent cross-contamination during the PCR assays including use of separate rooms for making reagent mixes, extraction of DNA, loading extracted DNA into reagent mix, and conducting the PCR process. Each work area had dedicated laboratory gowns, gloves, pipettes, and other supplies.

Colostrum samples were tested using similar methods as whole blood, with 2 exceptions. An initial centrifugation step of 10 min at 1,730 × *g* and 4°C to pellet cells and separate whey from lipid was done. Then 200 µL of whey and 200 µL of cells suspended in PBS solution were extracted separately to obtain DNA. The extraction methods were as described above using the KingFisher Flex Purification System (Thermo Fisher Scientific) with the MagMAX Core Nucleic Acid Purification Kit (Thermo Fisher Scientific), following the manufacturer's instructions. An additional extraction method with silica columns and centrifugation (DNeasy blood and tissue kit, Qiagen, Germantown, MD) was used following the manufacturer's instructions to obtain DNA from pelleted cells or whey. Because colostrum was a seldom used sample type for these PCR assays, a second extraction process was column based (QuickDNA Miniprep Plus Kit, Zymo Research) and done to determine if extraction method affected results of the PCR assays. Positive controls for the PCR assays included 2 mL of colostrum spiked with 100 µL of cattle blood known to be positive for bovine hemoplasma. The negative control was reagent mix without any additions.

Statistical analyses were performed using SAS version 9.4 (SAS Institute Inc., Cary, NC) and statistical significance was defined as *P* ≤ 0.05. Descriptive statistics were performed using PROC MEANS and used to characterize the participating animals. Normality of the data was evaluated using normal probability and box plots with PROC UNIVARIATE. The Mann–Whitney U test was used to evaluate the hypothesis that the age of the cow was associated with transplacental transmission. The cows were classified into 2 groups based on whether they delivered an infected calf or not, and the Mann–Whitney U test was employed to assess the hypothesis that transplacental transmission was associated with the PCR Ct.

The enrolled herd contained about 3,900 lactating cows, 474 preweaning calves, and 1,376 replacement heifers. All cows were housed in freestalls and gave birth in individual box stalls. Blood and colostrum samples were collected from Holstein cows (n = 39) and blood was collected from each calf (n = 39) immediately after birth, before ingestion of colostrum. The overall prevalence of hemoplasma infection in cows was 100% and all cows appeared clinically healthy. More specifically, 6 (15.3%, 95% CI: 4.0%–27.0%) cows were positive for *C. M. haemobos* only, whereas co-infection with *M. wenyonii* and *C. M. haemobos* was detected in 33 (84.6%, 95% CI: 73.2%–95.9%) cows (Table 1). Interestingly, none of the cows were infected solely with *M. wenyonii*. Of 39 calves, 4 (10.2%, 95% CI: 1.0%–20.0%) were positive for *C. M. haemobos* and all positive calves were born to a dam co-infected with both bovine hemoplasmas. No calves were positive for *M. wenyonii* (either single or as a co-infection). The mean age of cows was 2.9 ± 0.1 yr (SE) and ranged from 1.8 to 5.7 yr. The mean age of cows delivering an infected calf was numerically younger (2.4 ± 0.3 yr) than the age of cows who did not deliver an infected calf (3.0 ± 0.2 yr), but this difference was not significant (*P* = 0.48). The Ct values of the 4 calves born test positive for *C. M. haemobos* were 22.3, 27.1, 28.6, and 32.0. The mean Ct values for *C. M. haemobos* were 28.6 and 27.6 for cows that delivered test-positive and test-negative calves, respectively (*P* = 0.75).

**Table 1:** Apparent prevalence of infection with *Mycoplasma wenyonii*, *Candidatus Mycoplasma haemobos*, and co-infection tested using rt-PCR on blood samples collected from cows and their corresponding calves (before ingestion of colostrum) on 1 farm in Michigan in March 2023

Cow^2^	Calf^1^		Total (%)
	*C. M. haemobos* (%)	Negative (%)	
*C. M. haemobos* (%)	0 (0.0)	6 (100.0)	6 (15.3)
Co-infection (%)	4 (12.2)	29 (87.8)	33 (84.6)
Total (%)	4 (10.2)	35 (89.7)	39 (100.0)

1 There were no calves positive for *M. wenyonii* only or co-infected with both organisms.

2 There were no cows positive for *M. wenyonii* only, and all cows were positive for some organism.

The DNA for *M. wenyonii* or *C. M. haemobos* was not detected in any colostrum samples, but all of the spiked colostrum samples tested positive.

Infections in calves that were sampled immediately after birth were likely a result of transplacental transmission. Parturition was closely observed by researchers who collected blood immediately after birth (before ingestion of colostrum), thereby eliminating the possibility of other potential routes of infection. Transplacental transmission of hemoplasmas has been suggested for only a few species, including dogs (Lashnits et al., 2019), swine (Guimaraes et al., 2007), and alpacas (Almy et al., 2006). In cattle, several hemotropic organisms such as *Anaplasma marginale*, *Babesia bovis*, and *Theileria orientalis* are known to cause transplacental infection (Costa et al., 2016; Swilks et al., 2017); however, only limited research has evaluated the potential for vertical transmission of *M. wenyonii* and *C. M. haemobos*. The first evidence of transplacental transmission of *C. M. haemobos* was reported in Hungary by Hornok et al. (2011), and subsequent studies reported vertical transmission of *M. wenyonii* or *C. M. haemobos* (Sasaoka et al., 2015; Girotto-Soares et al., 2016; Niethammer et al., 2018). Our results support transplacental transmission of bovine hemoplasmas. The incidence of PCR-positive calves at birth in our investigation was similar to risks reported in Bavaria (2/25; 8.0%), Switzerland (4/38; 10.5%), and Brazil (4/22; 18.2%) (Niethammer et al., 2018; Hornok et al., 2011; Girotto-Soares et al., 2016, respectively), but less than reported in or Japan (4/17; 23.5%) (Sasaoka et al., 2015). Although we observed a high prevalence of hemoplasma infection in cows, the low proportion of calves born infected suggests that infection of cows is not the predominant pathway of infection. While the mechanisms by which hemoplasmas are transmitted through the placenta in cows are not known, for cattle infected with *A. marginale*, *B. bovis*, and *Babesia bigemina*, researchers have suggested that intrinsic characteristics of the organisms (such as their size and strain), as well as periparturient immunosuppression in dams, can increase subclinical infection, and might affect transmission through the placenta (Costa et al., 2016). Although the association between immunosuppression during the peripartum period and transplacental transmission of hemoplasmas requires further investigation, the adoption of management practices such as providing appropriate nutrition and effective stress management should be investigated as management strategies to mitigate potential risk associated with this mode of transmission.

In a previous random sampling cows on this farm (unpublished data), we found that of 16 adult cows, 3 were infected solely with *M. wenyonii*, 2 were PCR-positive for *C. M. haemobos*, and 9 were co-infected with both organisms. However, in the current study, none of the cows were found to be infected solely with *M. wenyonii*. In our companion study that tested calves and heifers twice over a 3- to 4-mo period (unpublished data), most heifers seemed to be persistent carriers, but a small proportion of animals appeared to have cleared the infection. Although the reasons for which animals appeared to clear the infection are not apparent, variations in the bacterial load of each hemoplasma may result in negative tests in previously positive animals. Despite variation in the prevalence of each hemoplasma, we previously established that hemoplasma infection is highly prevalent in this herd. In our companion study (unpublished data), we tested preweaning calves (from 1 to 60 d of age) for hemoplasmas, but none of the 31 calves tested on this farm were positive for infection. This contrasts with this study, where some newborn calves tested positive for hemoplasma. Based on our observation of transplacental transmission in this study, it is puzzling that all preweaning calves were negative in the earlier study. The 2 studies were conducted several months apart, and it is possible that a low prevalence of infection in preweaning calves reduced our ability to detect infection. However, the dynamics of infection with hemoplasmas in early life of calves is unknown and requires further investigation.

Similar to Hornok et al. (2011), all positive calves were born from a dam co-infected with both bovine hemoplasmas. Interestingly, all calves were solely infected with *C. M. haemobos* and infection with *M. wenyonii* was not detected. In previous studies, infection with *M. wenyonii* (Sasaoka et al., 2015) and co-infection with both organisms (Hornok et al., 2011) were reported in newborn calves. It is possible that variations in prevalence of hemoplasma organisms among dams could account for differences in hemoplasma infections in calves, but additional studies are needed to explore this possibility. Additional risk factors such as differences in breeds (Sasaoka et al., 2015; Niethammer et al., 2018), method of diagnosis, and geographic locations may affect the prevalence of hemoplasmas. Although our small study was not designed to establish associations between risk factors (such as age of the dam) and transplacental transmission, the numerical difference in age suggests that more research about age and risk of transplacental transmission should be performed. In a previous study that sampled mature cows (n = 2,521; Schambow et al., 2021), the apparent prevalence of hemoplasma infection was less in cows that were in parity 3+ as compared with primiparous cows but was not associated with stage of lactation. This observation may suggest that the concentration of bacteria in blood is greater in younger cows, thus facilitating transplacental transmission. However, a larger study that enrolls cows from multiple herds is needed to better characterize potential relationships between age and risk of transplacental transmission of hemoplasmas. The sex of the infected calves (2 females, 2 males) did not appear to influence the risk of infection. Similar findings related to the age of the cow and the sex of the calves have been previously reported (Hornok et al., 2011).

In addition to transplacental transmission of hemoplasmas, other modes of transmission to calves should be considered. During the prepartum period, there is an increased permeability of the mammary gland, which potentially allows erythrocytes to be secreted into colostrum (McGrath et al., 2016). The ingestion of colostrum containing infected red blood cells could be a possible route of transmission from dams to calves; however, this route has not been confirmed. Our study is the first to investigate the presence of DNA of both *C. M. haemobos* and *M. wenyonii* in colostrum of dairy cows, but we failed to detect hemoplasmas in colostrum using analysis based on 16S rRNA gene. A study using colostrum from beef cattle analyzed ribonuclease P RNA gene for *M. wenyonii* using 17 colostrum samples from infected dams but did not detect any infected colostrum (Sasaoka et al., 2015). In our previous study using the same farm (de Souza Ferreira et al., 2024), a small proportion of preweaning calves were positive for hemoplasmas, but all of these calves had ingested colostrum. This suggests that even if hemoplasmas are present in the colostrum and can potentially serve as a source of infection for calves, their contribution to infections in calves appears to be very low. Detecting such a small prevalence would likely require a larger sample size. Another possible explanation for the absence of hemoplasmas in the colostrum tested could be related to negative samples not containing blood from the dams. Hammer et al. (2016) reported that colostrum can contain significant quantities of *T. orientalis* as determined by qPCR but this might not be the case for hemoplasmas. Moreover, other factors such as the bacterial load of hemoplasmas in colostrum may have been too low to be detected, or it is possible that red blood cells infected with bovine hemoplasmas cannot be transmitted to colostrum. Despite these considerations, it is important to acknowledge that our study was conducted on a single farm, and it is possible that unknown farm-level factors could affect the transmission of this organism. Additional research is needed to investigate the role of colostrum in transmission of hemoplasmas, but our result suggests that colostrum might not be involved in the transmission of hemoplasmas.

Our results suggest that transplacental transmission could serve as an alternative route of bovine hemoplasma transmission. However, the low prevalence of infected calves at birth indicates that this pathway plays a minor role in infection. Additionally, hemoplasma do not appear to be present in colostrum.
